# Where Are the Women? Exploring the 14-Fold Gender Gap in Tunisian Addiction Services

**DOI:** 10.1192/j.eurpsy.2026.11956

**Published:** 2026-07-31

**Authors:** E. M. Bahri, J. Nasri, S. Hleli, M. Bennsir, W. Abdelghaffar

**Affiliations:** Hope Department for Addiction Care, Jbel Ouest Health Center, Zaghouan, Tunisia

## Abstract

**Introduction:**

Substance use disorders (SUDs) are a major global health challenge, heavily influenced by gender. Women with SUDs face unique barriers—such as stigma, fear of losing child custody, and co-morbid psychiatric disorders—that limit their access to treatment, creating a significant care gap.

**Objectives:**

This study aimed to quantify the gender-based treatment gap and profile the patient population of a Tunisian addiction service to identify barriers and inform the development of gender-sensitive care strategies.

**Methods:**

This single-center, retrospective, cross-sectional study was conducted at the Hope Department for Addiction Care at the Jbel Ouest Health Center in Tunisia. Data from all 907 patients who presented for consultation between January 2023 and June 2025 were included. Demographic and clinical variables—including gender, marital status, education, employment, personal history, and primary substance—were retrospectively collected from electronic medical records and anonymized. Data were analyzed using IBM SPSS Statistics (Version 27), with descriptive statistics presented as frequencies and percentages.

**Results:**

Analysis revealed a dramatic gender imbalance in service utilization. From a total of 907 patients, 845 (93.2%) were male and only 62 (6.8%) were female, indicating that men sought treatment at a rate nearly 14 times higher than women.

The analysis revealed a mean age of 30.41 years (SD = 9.61), with an age range from 13 to 65 years.

The typical patient was most likely to be Single (73.5% of valid responses) and to have attained a Secondary level of education (74.5%). The employment status distribution pointed towards significant socioeconomic precarity, with the largest groups being Day Laborers (36.5%) and Unemployed individuals (34.6%).

A history of Personal Psychiatric History was the most frequently reported antecedent (46.9%). Cannabis was the predominant Primary Substance of abuse by a large margin (60.3%), followed by Cocaine (11.8%) and Alcohol (7.5%).

**Conclusions:**

The extreme under-representation of women suggests significant systemic and sociocultural barriers beyond prevalence rates. These findings highlight an urgent need to develop targeted, gender-sensitive public health strategies and treatment paradigms to ensure equitable access to addiction care in Tunisia.

**Disclosure of Interest:**

None Declared

